# Antimicrobial resistance profiling and molecular subtyping of *Campylobacter *spp. from processed turkey

**DOI:** 10.1186/1471-2180-9-203

**Published:** 2009-09-21

**Authors:** Ellen M Lutgen, John M McEvoy, Julie S Sherwood, Catherine M Logue

**Affiliations:** 1 Department of Veterinary and Microbiological Sciences, North Dakota State University, Fargo, ND, USA

## Abstract

**Background:**

*Campylobacter *is a major cause of human disease worldwide and poultry are identified as a significant source of this pathogen. Most disease in humans is associated with the consumption of contaminated poultry or cross-contamination with other foods. The primary drugs of choice for treatment of human campylobacteriosis include erythromycin and ciprofloxacin. In this study, we investigated the prevalence of resistance to erythromycin and ciprofloxacin in *Campylobacter *isolates recovered from turkey carcasses at two processing plants in the Upper Midwest US. Further analysis of a subset of isolates was carried out to assess resistance and genotype profiles.

**Results:**

*Campylobacter *isolates from plant A (n = 439; including 196 *C. coli *and 217 *C. jejuni*) and plant B (n = 362, including 281 *C. coli *and 62 *C. jejuni*) were tested for susceptibility to ciprofloxacin and erythromycin using agar dilution. *C. coli *were more frequently resistant than *C. jejuni *in both plants, including resistance to ciprofloxacin (28% of *C. jejuni *and 63% of *C. coli*, plant B; and 11% of *C. coli*, plant A). Erythromycin resistance was low among *C. jejuni *(0% plant A and 0.3% plant B) compared to *C. coli *(41%, plant A and 17%, plant B). One hundred resistant and susceptible isolates were selected for additional antimicrobial susceptibility testing, restriction fragment length polymorphism analysis of the *flaA *gene (*fla *typing), and pulsed-field gel electrophoresis (PFGE). *Fla*-PFGE types obtained (n = 37) were associated with a specific plant with the exception of one type that was isolated from both plants. *C. coli *isolates (n = 65) were grouped into 20 types, while *C. jejuni *isolates (n = 35) were grouped into 17 types. Most isolates with identical *fla-*PFGE patterns shared identical or very similar antimicrobial resistance profiles. PFGE alone and composite analysis using *fla-*PFGE with resistance profiles separated *C. jejuni *and *C. coli *into distinct groups.

**Conclusion:**

Ciprofloxacin and erythromycin resistance in *Campylobacter *recovered from processed turkey occurred more frequently among *C. coli *than *C. jejuni*. *Fla*-PFGE types were associated with a particular species, antimicrobial resistance profiles, and a specific plant. Molecular subtyping in this study provided more information about the relationships among antimicrobial-resistant *Campylobacter *at the processing level.

## Background

*Campylobacter *spp. are one of the major causes of human gastroenteritis worldwide and are estimated to cause over two million cases of illness annually in the U.S. [1]. Greater than 95% of human infections are due to *C. jejuni *or *C. coli *[2]. Human disease is characterized by diarrhea, fever, and abdominal cramping [3]. Campylobacteriosis is most often associated with the handling and consumption of raw or undercooked poultry [2-4].

In poultry, *Campylobacter *is considered a commensal organism [4]. When colonized poultry enter the processing plant, contamination of the carcass and processed product can result [4]. Turkey is an important reservoir of *Campylobacter*; studies have reported prevalence rates of 65-95% in U.S. turkeys at production [5-7]. In a study from our lab, the prevalence of *Campylobacter *was 34.9% from two turkey processing plants [8], while at the retail level, the organism has been detected in 1.0-15% of samples tested [9,10].

Human campylobacteriosis is generally self-limiting, although in severe cases it requires antimicrobial therapy. Erythromycin and ciprofloxacin are often the drugs of choice [11]. Fluoroquinolones such as ciprofloxacin have been used for first-line treatment of bacterial gastroenteritis in the absence of a microbiological diagnosis [3]. However, an increase in fluoroquinolone-resistant *Campylobacter *infections in humans has been documented worldwide [12-14], and may be associated with fluoroquinolone use in food animals [12,15,16]. Although the approval of enrofloxacin (a fluoroquinolone) for use in poultry was withdrawn by the U.S. Food and Drug Administration in 2005, it is possible that fluoroquinolone-resistant *Campylobacter *will persist in poultry flocks [17]. Macrolides such as erythromycin have been the preferred treatment for *Campylobacter *infections [3,13]; however, increasing resistance to erythromycin among *Campylobacter *has been documented, particularly in *C. coli *[12,18-20]. The duration of illness, risk of invasive illness, or poorer treatment response has been shown to be greater for patients infected with quinolone- or macrolide-resistant *Campylobacter *[16,21-23]; although Wassenaar et al. [24] did not find these effects associated with fluoroquinolone-resistant *Campylobacter *infections. In *Campylobacter*, resistance to quinolones and macrolides is primarily associated with mutations in the *gyrA *and 23S rRNA genes, respectively [20,25]. The involvement of the CmeABC multidrug efflux pump in resistance to both classes of antimicrobials has also been recognized [26,27].

Information about antimicrobial resistance of *Campylobacter *at different levels of production is important for the development of control strategies for this pathogen. In addition, differentiation of antimicrobial-resistant strains is necessary to investigate the epidemiology of resistance. Restriction fragment length polymorphism (RFLP) analysis of the *flaA *gene (*fla *typing) and pulsed-field gel electrophoresis (PFGE) are two genotyping methods used to successfully differentiate *Campylobacter *strains [28,29]. This study was conducted to assess the ciprofloxacin and erythromycin resistance in *Campylobacter *isolated from turkey at the processing level. *Fla *typing, PFGE, and antimicrobial susceptibility testing were used to characterize a subset of ciprofloxacin- and/or erythromycin-resistant and susceptible *Campylobacter *isolates obtained from pre and post chill turkey carcasses and chill water.

## Results

### Antimicrobial susceptibility testing

**Figure 1:**
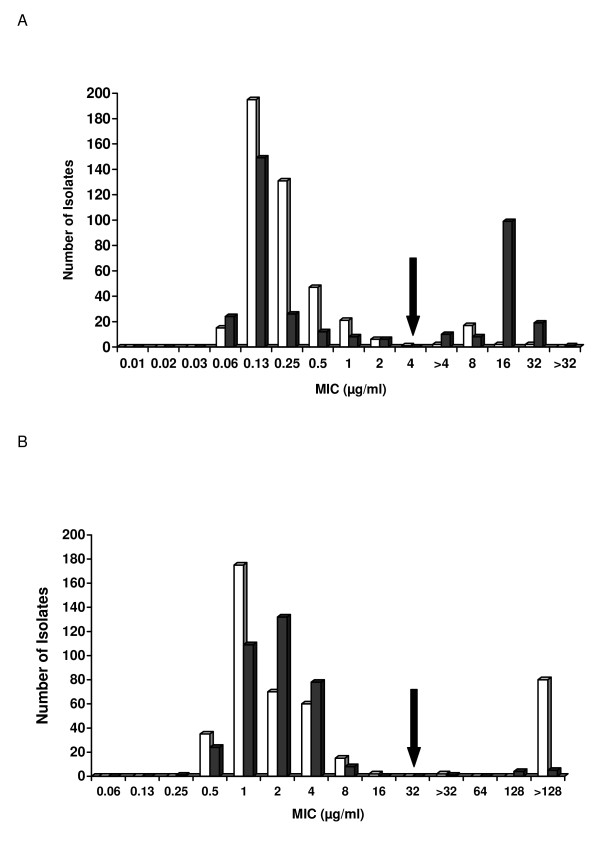
**Antimicrobial susceptibility profiles of *Campylobacter *isolates (n = 801)**. Isolates from plant A (n = 439; open bars) and plant B (n = 362; black bars) were tested for antimicrobial susceptibility using agar dilution. **A**. The frequency of MICs obtained for ciprofloxacin. The arrow denotes the breakpoint of ≥ 4 μg/ml. **B**. The frequency of MICs obtained for erythromycin. The arrow denotes the breakpoint of ≥ 32 μg/ml.

Figure 1A and 1B shows the MICs of 801 *Campylobacter *isolates to ciprofloxacin and erythromycin. Few isolates were co-resistant to both antimicrobials (2 from plant A [0.45% of plant A isolates] and 9 from plant B [2.5% of plant B isolates]). Resistant isolates were recovered from carcasses at pre chill and post chill at both plants. No significant difference (*P *> 0.01) was observed between the number of ciprofloxacin-resistant or erythromycin-resistant isolates obtained from either process stage at plant A (Table 1).

**Table 1:** Antimicrobial resistance and sampling stage distribution of *Campylobacter *isolates (n = 801).

		Plant A			Plant B	
Sampling Stage	Total Isolates	Ciprofloxacin Resistance	Erythromycin Resistance	Total Isolates	Ciprofloxacin Resistance	Erythromycin Resistance
Pre Chill	225^*a *^(51)^*b*^	7^*c *^(3.1)^*d*^	46^*c *^(20)^*d*^	242^*a *^(67)^*b*^	99^*c *^(41)^*d*^	6^*c *^(2.5)^*d*^
Post Chill	209 (48)	16 (7.7)	35 (17)	119 (33)	37 (31)	4 (3.4)
Chill Water	5 (1.1)	1 (20)	1 (20)	1 (0.3)	1 (100)	0 (0)

Total	439	24^*c *^(5.5)^*e*^	82^*c *^(19)^*e*^	362	137^*c *^(38)^*e*^	10^*c *^(2.8)^*e*^

^*a*^Number of total isolates tested.

^*b*^Percentage of total isolates tested.

^*c*^Number of isolates resistant.

^*d*^Percentage of isolates resistant among total tested for that stage.

^*e*^Percentage of isolates resistant among total tested for that plant.

Differences were observed in the frequency of resistance among *C. coli *compared to *C. jejuni *(Table 2). *C. coli *were more likely to be erythromycin-resistant compared to *C. jejuni *(41% plant A and 17% plant B compared to 0.0%, plant A and 0.3%, plant B) (*P *< 0.01). *C. coli *were also more likely to be ciprofloxacin-resistant compared to *C. jejuni *in both plant A (*C. coli*, 11%; *C. jejuni*, 0.0%) and plant B (*C. coli*, 63%; *C. jejuni*, 28%) (*P *< 0.01).

**Table 2:** Ciprofloxacin and erythromycin resistance of *Campylobacter *spp. from two commercial turkey processing plants.

		Plant A			Plant B	
**Species**	**No. (%)**	**No. (%) resistant to ciprofloxacin**	**No. (%) resistant to erythromycin**	**No. (%)**	**No. (%) resistant to ciprofloxacin**	**No. (%) resistant to erythromycin**
*C. jejuni*	217^*a *^(49)^*b*^	0^*c *^(0.0)^*d*^	0^*c *^(0.0)^*d*^	281^*a *^(78)^*b*^	80^*c *^(28)^*d*^	1^*c *^(0.3)^*d*^
*C. coli*	196 (45)	22 (11)	81 (41)	62 (17)	39 (63)	9 (17)
*C. fetus*	1 (0.2)	0 (0.0)	0 (0.0)	3 (0.8)	3 (100)	0 (0.0)
*C. lari*	7 (1.6)	2 (29)	1 (14)	0 (0.0)	n/a	n/a
*C. upsaliensis*	3 (0.7)	0 (0.0)	0 (7.0)	0 (0.0)	n/a	n/a
*Campylobacter *spp.	15 (3.4)	0 (0.0)	0 (0.0)	16 (4.4)	15 (94)	0 (0)

Total	439	24^*c *^(5.5)^*e*^	82^*c *^(19)^*e*^	362	137^*c *^(38)^*e*^	10^*c *^(2.8)^*e*^

^*a*^Number of total isolates tested.

^*b*^Percentage of total isolates tested.

^*c*^Number of isolates resistant.

^*d*^Percentage of isolates resistant among total tested for that species.

^*e*^Percentage of isolates resistant among total tested for that plant.

**Figure 2:**
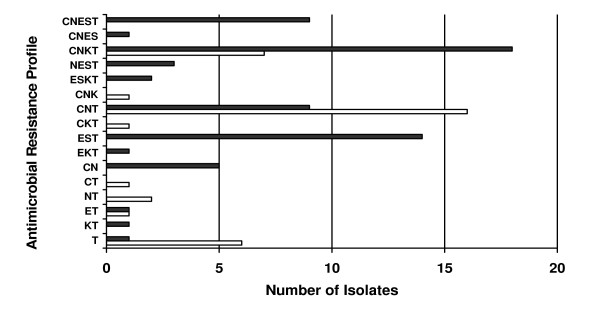
**Antimicrobial resistance profiles and frequency among selected *Campylobacter *isolates (n = 100)**. *C. jejuni *(n = 35; open bars) and *C. coli *(n = 65; black bars) isolates were tested for antimicrobial resistance using agar dilution. Presence of a letter indicates resistance, with C = ciprofloxacin, N = nalidixic acid, E = erythromycin, S = streptomycin, K = kanamycin, and T = tetracycline.

Additional antimicrobial susceptibility testing conducted on a subset of isolates selected for subtyping (n = 100) found that isolates from plant A (n = 51; *C. jejuni*, 8; *C. coli*, 43) were resistant to tetracycline (100%), nalidixic acid (49%; *C. jejuni*, 2; *C. coli*, 23), kanamycin (41%; *C. jejuni*, 0; *C. coli*, 21), and streptomycin (41%; *C. jejuni*, 0; *C. coli*, 21), while those from plant B (n = 49; *C. jejuni*, 27; *C. coli*, 22) were resistant to nalidixic acid (94%; *C. jejuni*, 24; *C. coli*, 22), tetracycline (86%; *C. jejuni*, 26; *C. coli*, 16), kanamycin (20%; *C. jejuni*, 9; *C. coli*, 1) and streptomycin (18%; *C. jejuni*, 0; *C. coli*, 9). Sixteen different drug resistance profiles were identified, with most isolates displaying resistance to more than one agent (Figure 2). None of the isolates were resistant to all six agents tested. The two most prevalent multiple resistance profiles observed were 1) ciprofloxacin, nalidixic acid and tetracycline for 25 isolates (most common profile among *C. jejuni*) and 2) ciprofloxacin, nalidixic acid, kanamycin and tetracycline for 25 isolates (most common profile among *C. coli*)

### *Fla *typing and pulsed-field gel electrophoresis

**Figure 3:**
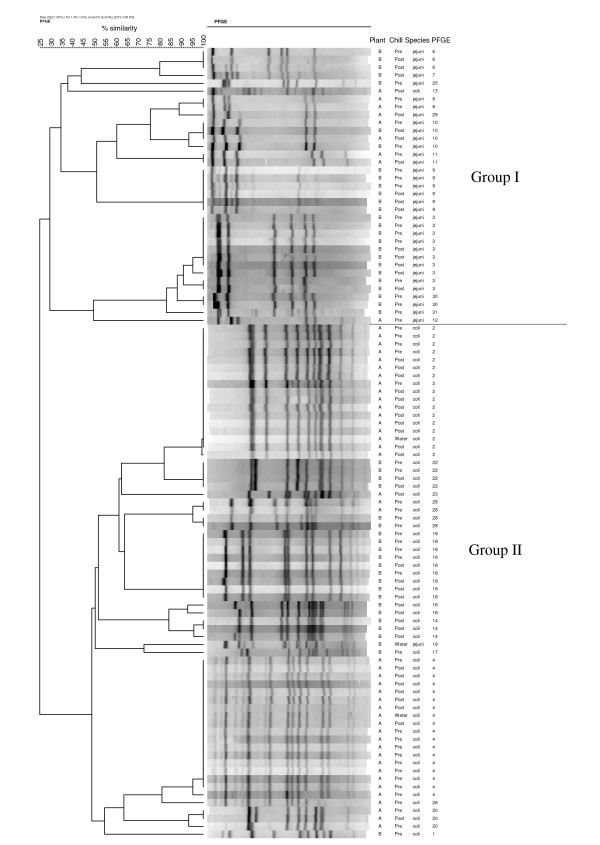
**Dendrogram of PFGE types for *Campylobacter *isolates (n = 100)**.

**Figure 4:**
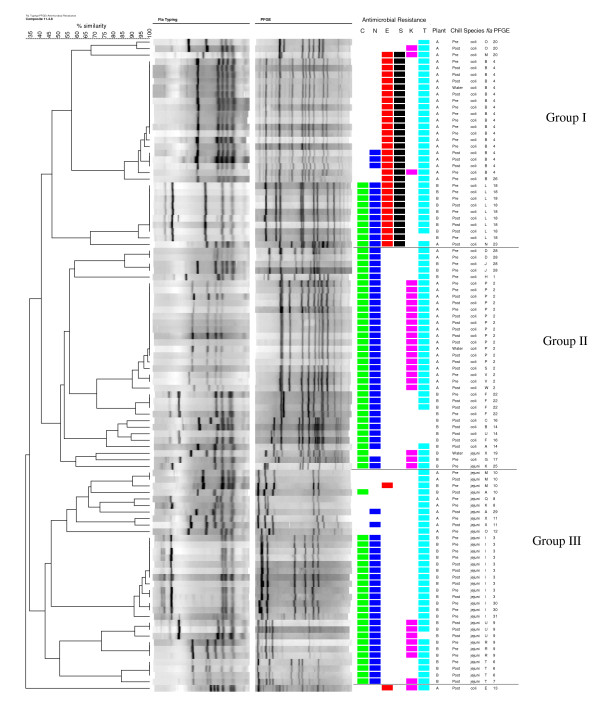
**Composite dendrogram for *Campylobacter *isolates (n = 100) based on *fla *typing, PFGE, and antimicrobial resistance**. Presence of a colored square indicates resistance, with C = ciprofloxacin, N = nalidixic acid, E = erythromycin, S = streptomycin, K = kanamycin, and T = tetracycline.

All of the isolates examined (n = 100) tested positive for the *flaA *gene and 24 different *fla *types were observed. Twenty-six PFGE types were observed. *Fla *typing separated the isolates into three major groups at 50% similarity (data not shown), while PFGE separated them into two major groups at 30% similarity (Figure 3). Similar *fla *types were found in isolates originating from different plants (types A, B, K, M and X). Two PFGE types were detected in isolates from both plants (types 10 and 28). Thirty-seven combined *fla-*PFGE types were obtained, 22 of which contained only single isolates (Figure 4). Plant A isolates were grouped into 16 *fla-*PFGE types and plant B isolates were grouped into 22 *fla-*PFGE types. *Fla*-PFGE types were unique to a particular plant with the exception of M10, which was isolated from both plants on different days in the same month. M10 was also isolated once from plant A in the previous month. In both plants, some isolates obtained from different sampling stages (pre or post chill) had identical *fla*-PFGE types.

Six *fla *types were observed for *C. jejuni *isolates, while fourteen *fla *types were observed for *C. coli *isolates. Four *fla *types within two of the three major clusters included isolates of *C. jejuni *and *C. coli *(data not shown). Using PFGE, *C. jejuni *isolates were divided into 13 PFGE types, while *C. coli *were also divided into 13 PFGE types. The two major clusters obtained with PFGE generally separated the two species (Figure 3). Combined *fla-*PFGE types were unique to a particular species. *C. coli *isolates (n = 65) were grouped into 20 *fla-*PFGE types; three of these *fla-*PFGE types (B4, L18, and P2) contained 62% of the total *C. coli *isolates. *C. jejuni *isolates (n = 35) were grouped into 17 *fla-*PFGE types; one *fla-*PFGE type (I3) contained 29% of the *C. jejuni *isolates, while the other *fla-*PFGE types included no more than 3 *C. jejuni *isolates each.

Antimicrobial resistance profiles and combined *fla-*PFGE types are shown in Figure 4. Thirty-seven isolates with the same *fla-*PFGE type had identical resistance profiles, including *fla-*PFGE types J28, D28, I30, I3, P2, V2, R9, and T6. Forty-one isolates with the same *fla-*PFGE type had either identical resistance profiles or very similar resistance profiles, including *fla*-PFGE types B4, U9, F22, L18, M10, X11, and O20. Within some *fla-*PFGE types, the MICs for the antimicrobials varied, generally between one to four dilutions (data not shown). On occasion, different *fla-*PFGE types from the same plant had identical antimicrobial resistance profiles (Figure 4). For example, types A14 and J28 from plant B were both resistant to ciprofloxacin, nalidixic acid, and tetracycline.

Composite analysis (Figure 4) using *fla *typing, PFGE, and antimicrobial resistance profiles separated the isolates into 30 distinct types. At 43% similarity, three major clusters (I, II, and III) were evident. One isolate was not clustered into any of these three groups. The majority of isolates in group II were *C. coli*, while all of the isolates in groups I and III were *C. coli *and *C. jejuni*, respectively.

The numerical index of discrimination (*D*) was used to evaluate the results of *fla *typing, PFGE, and antimicrobial resistance profiling. The discrimination index was highest for *fla-*PFGE analysis (*D *= 0.9321) followed by PFGE (*D *= 0.9147), composite data (all three methods, *D *= 0.9137), *fla *typing (*D *= 0.9119), and antimicrobial resistance profiling (*D *= 0.8430).

## Discussion

*Campylobacter *isolates from two turkey processing plants in the upper Midwest were examined for susceptibility to ciprofloxacin and erythromycin, antimicrobial agents used for the treatment of human campylobacteriosis. Although co-resistance to both antimicrobials was low, resistance was detected and differences were observed in the frequency of resistance in relation to species. *C. coli *from plant A (41%) and plant B (17%) were more likely to be erythromycin-resistantcompared to *C. jejuni *(plant A, 0.0%; plant B, 0.3%) (*P *< 0.01). Similarly, other studies on *Campylobacter *isolated from poultry have reported that erythromycin resistance occurs more frequently in *C. coli *than *C. jejuni *[6,9,18,30-32]. The occurrence of erythromycin resistance among *C. coli *isolated from the processing environment in this study (41%, plant A and 17%, plant B) was greater in comparison to 11.8% and 12.5% for *C. coli *from retail turkey in the U.S. [9] and Germany [33], respectively. Erythromycin resistance among *C. jejuni *in this study was very low, similar to the aforementioned reports on retail turkey where resistance was 0% for *C. jejuni *in both countries [9,33]. In contrast, 6.4% of *C. jejuni *obtained from turkeys at a Belgian slaughterhouse were resistant [32].

In this study, the frequency of ciprofloxacin resistance was also found to be higher in *C. coli *(plant A, 11%; plant B, 63%) compared to *C. jejuni *(plant A, 0.0%; plant B, 28%) (*P *< 0.01). Others have reported a higher occurrence of fluoroquinolone resistance in *C. coli *compared to *C. jejuni *as well [6,19,30,34]. In comparison to previous studies conducted at different parts of the production system, ciprofloxacin resistance at plant B (28% in *C. jejuni *and 63% in *C. coli*) was similar to U.S. turkeys at the farm level [6,35], Belgian turkey at slaughter [32] and retail turkey in Germany [33].

Resistance to multiple antimicrobial agents was observed in most of the *Campylobacter *isolates selected for molecular profiling (Figures 2 and 4). Most isolates were resistant to 3 or 4 agents. The most frequent resistance profile observed among *C. jejuni *isolates was to ciprofloxacin, nalidixic acid, and tetracycline. This profile was also reported as the most common multidrug resistance pattern for human *Campylobacter *isolates received through NARMS from 1997-2001 [13]. In this study, the most common multiple resistance pattern among *C. coli *isolated from turkey was resistance to ciprofloxacin, nalidixic acid, kanamycin, and tetracycline. These findings differ from reports by Lee et al. [36] and Luangtongkum et al. [6], where resistance profiles of ciprofloxacin, nalidixic acid, erythromycin, streptomycin, kanamycin, and tetracycline resistance predominated in *C. coli *from turkeys.

In addition to expanded antimicrobial resistance testing, *fla *typing and PFGE were used to further characterize antimicrobial-resistant *C. jejuni *and *C. coli *from processed turkey. It was observed that most of the *Campylobacter *isolates with identical *fla-*PFGE types had the same antimicrobial resistance profiles, a finding also noted by Ge et al. using PFGE [30]; however, analysis of additional antimicrobial-sensitive strains would be indicated. For subtyping *C. jejuni *and *C. coli *in this study, the greatest discrimination index was obtained using *fla-*PFGE together. Similarly, Nayak et al. [35] found a combination of subtyping methods for *Campylobacter *isolated from turkey farms had a greater discriminatory value than a single method. In the current study, *fla *typing failed to distinguish completely between the two *Campylobacter *species, a finding also noted by others [37-39]. In contrast, PFGE showed greater discrimination in separating the two species, which can be attributed to its ability to detect whole genome restriction site polymorphisms [29]. In addition to discriminatory value, other characteristics of these molecular typing methods should be acknowledged, which have been reviewed elsewhere [28,29,37,40,41]. *Fla *typing is a useful tool for subtyping campylobacters [39,42], and has the advantages of being simple, quick, and low cost [28,29,42]. Nayak et al. reported that *fla *typing was more suitable than PFGE for typing *C. coli *isolated from turkey farms [35]. However, the potential for recombination within the *fla *genes is a drawback of using *fla *typing alone or for long-term studies [29,43]. For this reason, and because *fla *typing is generally less discriminatory than PFGE, it is recommended to use *fla *typing in conjunction with other typing methods [29,41]. PFGE is highly discriminatory and well-accepted for typing campylobacters, although it is laborious and can be expensive [29,37]. PFGE profiles may also be affected by genetic instability in *Campylobacter *[28,29].

In this study, the genetic diversity of antimicrobial-resistant strains varied between *C. coli *and *C. jejuni*. One *fla-*PFGE type (I3) contained 29% of the *C. jejuni *isolates while the remaining 16 *fla-*PFGE types contained one to three isolates each. In contrast, most of the *C. coli *isolates (62%) were grouped into only three *fla-*PFGE types, suggesting less diversity among *C. coli*. Bae et al. [44] demonstrated that PFGE types of antimicrobial-resistant *C. coli *from cattle were less diverse than those of *C. jejuni*, and Nayak et al. [35] reported a similar effect among antimicrobial-resistant *C. coli *and *C. jejuni *from turkey farms. Wesley et al. [7] described the opposite case, that *C. coli *from turkeys were more diverse than *C. jejuni *based on PFGE, although antimicrobial resistance was not determined.

The *Campylobacter *isolates examined in this study originated from turkey carcasses at either the pre or post chill stages of processing. The prevalence of ciprofloxacin or erythromycin resistance was similar from either stage in plant A. In contrast, Berrang et al. found that the numbers of erythromycin-resistant *C. jejuni *on broiler carcasses were reduced after chilling, and suggested further study to determine whether this resistance influences the ability of *Campylobacter *to endure immersion chilling [45]. In the current study, several of the same *fla-*PFGE types were recovered from both stages, indicating that some ciprofloxacin- and/or erythromycin-resistant strains were present beyond chilling. Information about antimicrobial-resistant *Campylobacter *on post-chill turkey product is limited and further study is needed.

Most of the *fla-*PFGE types (36 of 37) in the current study were unique to a particular plant. Similarly, Rasschaert et al. [46] demonstrated that most *fla-*PFGE types obtained from broilers at three processing plants were unique within a particular plant. The two plants participating in the current study were located approximately 150 miles apart in different states and were not likely to receive turkeys from the same farms. Isolation of the same *fla-*PFGE type (M10) from both plants may suggest a common source of this type, and warrants further investigation. However, it must be noted that the isolates subtyped for this study comprised a small portion of the entire *Campylobacter *collection (n = 801) tested, which may influence the frequency of *fla*-PFGE types obtained and is a limitation of our study.

Clustering using PFGE alone or *fla-*PFGE in conjunction with resistance profiles separated *C. jejuni *and *C. coli *into different groups. The diversity of genetic profiles, in conjunction with differences in resistance profiles by species, further supports the importance of considering *C. jejuni *and *C. coli *separately in epidemiological investigations [7,30,47,48]. Although *C. jejuni *is implicated in most campylobacteriosis cases, human illness attributed to *C. coli *is also recognized [13,47,49,50]. *C. coli *is often associated with pigs; but was prevalent in turkeys in our previous study [8] and those of others [7,51]. In Denmark, poultry, but not pigs, were associated with human *C. coli *infections [48].

## Conclusion

This study found that ciprofloxacin and erythromycin resistance was present in *Campylobacter *recovered from processed turkey in the Upper Midwest, and the prevalence differed significantly between *C. jejuni *and *C. coli*. Resistance observed in these strains has the potential to complicate the effectiveness of treatment for poultry-acquired *Campylobacter *infections in humans should they remain on the processed product. Molecular subtyping using *fla *typing and PFGE provided additional information on antimicrobial-resistant *Campylobacter *from processed turkey. *Fla-*PFGE types were relatively diverse and associated with a specific plant and species. Some ciprofloxacin and/or erythromycin resistant isolates with the same *fla-*PFGE types were recovered from processing both before and after chilling. Factors contributing to the occurrence of antimicrobial-resistant *Campylobacter *in processed turkey warrant further investigation.

## Methods

### Campylobacter isolates

*Campylobacter *isolates in this study (n = 801, Table 2) were obtained from two unrelated Midwestern processing plants (A and B) prior to the FDA ban of enrofloxacin use in poultry [8]. Plant A received turkeys from independent producers belonging to a farmers' cooperative, while plant B received turkeys from producers under contract with a large turkey processing company. Isolates were recovered and identified by Logue et al. as previously described [8]. Briefly, isolates were recovered from whole carcass swabs collected from randomly selected carcasses at two points on the processing line: pre chill and post chill, from plants visited monthly over a period of 12 months [8]. Samples of the chill water were also collected. Birds sampled on a single day were usually from one supplier or farm. Throughout all parts of the study, isolates were removed from -80°C storage in *Brucella *broth (Becton Dickinson, Cockeysville, Md.) with 20% glycerol and cultured onto sheep blood agar (BBL Prepared Media Trypticase Soy Agar II, 5% Sheep Blood; Becton Dickinson, Sparks, Md.). All cultures were incubated in a microaerobic environment of approximately 14% CO_2 _and 6% O_2 _generated by Pack-Micro Aero (Mitsubishi Gas Chemical, New York, N.Y.).

### Antimicrobial susceptibility testing

Antimicrobial susceptibility testing on all isolates (n = 801) was conducted using the agar dilution method [52,53] with testing ranges of 0.008-4 μg/ml for ciprofloxacin (Serologicals Proteins, Kankakee, Ill.) and 0.06-32 μg/ml for erythromycin (Sigma Chemical, St. Louis, Mo.). *C. jejuni *ATCC #33560 was used as a quality control strain [11,53]. Resistance breakpoints were ≥ 4 μg/ml for ciprofloxacin and ≥ 32 μg/ml for erythromycin [54]. Isolates (n = 241) with an MIC of > 4 μg/ml for ciprofloxacin and/or an MIC of > 32 μg/ml for erythromycin were re-tested with extended antimicrobial concentrations of 0.5-32 μg/ml for ciprofloxacin and 2.0-128 μg/ml for erythromycin.

One hundred isolates (n = 51, plant A and n = 49, plant B) were selected for further characterization. A similar number of isolates from each plant and processing stage were included in the subset, comprised of 58 ciprofloxacin-resistant isolates, 22 erythromycin-resistant isolates, 10 co-resistant isolates, and 10 isolates sensitive to these two antimicrobials. *C. jejuni *and *C. coli *species identification was confirmed using multiplex PCR as described previously [55]. Testing for susceptibility against tetracycline, streptomycin, kanamycin and nalidixic acid was conducted using the agar dilution method [52,53]. The test ranges used were 0.06-32 μg/ml for tetracycline (Sigma), 0.125-64 μg/ml for streptomycin (Sigma) and kanamycin (Amresco, Solon, Ohio), and 0.25-128 μg/ml for nalidixic acid (Sigma). The quality control strain used was *C. jejuni *ATCC #33560 [11,53]. For streptomycin and kanamycin testing, *Escherichia coli *ATCC #25922 and *C. jejuni *ATCC #33560 were included. *Campylobacter *isolates were defined as resistant or sensitive based on breakpoints of ≥ 16 μg/ml for tetracycline, ≥ 64 μg/ml for nalidixic acid, and ≥ 64 μg/ml for streptomycin and kanamycin [54,56].

### *Fla *typing

*Fla *typing (n = 100) was carried out using the method of Nachamkin et al. [57] with minor modifications. Whole cell lysate [58] was used as the template. PCR amplification was performed in a Mastercycler gradient 5331 thermocycler (Eppendorf, Hamburg, Germany). *C. jejuni *ATCC #700819 was used as the positive control, and sterile water was substituted for the DNA template as the negative control. To confirm the presence of the 1.7 kb *flaA *amplicon, 10 μl of the PCR product was subjected to gel electrophoresis followed by ethidium bromide staining and UV transillumination. *Dde*I (Promega, Madison, Wis.) was used to digest 5 μl of the *flaA *PCR product according to the manufacturer's instructions at 37°C for 12-16 h overnight. Digested samples were electrophoresed on a 2% agarose gel, followed by staining in 0.5 μg/ml ethidium bromide solution and UV transillumination. A 100 bp ladder (Promega) was used as a molecular size standard.

### Pulsed-field gel electrophoresis

Pulsed-field gel electrophoresis (PFGE) was performed using the PulseNet method [59] with slight modifications. *Salmonella enterica *serotype Braenderup H9812 (ATCC #BAA-664) was used as the molecular weight size standard. Restriction digestion of each sample plug slice was carried out in a 100 μl mixture containing 85 μl sterile water, 10 μl 10× J buffer, 4 μl of 10 U/μl *Sma*I (Promega), and 1 μl BSA at 25°C for 3 h. Electrophoresis was performed using the Chef Mapper system (Bio-Rad, Hercules, Calif.) and the following conditions: auto algorithm function (50 kb low molecular weight and 400 kb high molecular weight), run time 18 h, initial switch time 6.76 s and final switch time 38.35 s. Gels were stained with 1 μg/ml ethidium bromide solution for 30 min, destained in 500 ml reagent grade water for 60-90 min with water changes every 20 min, and viewed under UV transillumination.

### Documentation and analysis of *fla *typing and PFGE patterns

Gels were photographed and recorded as digital TIFF images using an Alpha-Innotech imager (Alpha Innotech, San Leandro, Calif.). Images were analyzed with Fingerprinting II Informatix software (Version 3.0, Bio-Rad). Band matching and cluster analysis was performed using an unweighted pair group method with arithmetic averages (UPGMA) and the Dice coefficient with 1% optimization and tolerance levels. Based on the dendrogram obtained from the cluster analysis, letters were assigned to designate *fla *types and numbers were assigned to designate PFGE types. Isolates with > 90% similarity were assigned to the same *fla *type or PFGE type. Composite cluster analysis including *fla *typing, PFGE, and antimicrobial resistance testing data was performed using the Fingerprinting II Informatix software. The composite dendrogram was determined by UPGMA using the average from the experiment as a coefficient for similarity and correction for internal weights.

### Statistical analysis

The χ^2 ^test was used to analyze the significance of the difference between ciprofloxacin and erythromycin resistance rates, including *C. jejuni *compared to *C. coli *in each plant, and pre chill compared to post chill in plant A. An α of 0.01 was used for statistical significance.

The discriminatory ability of *fla *typing, PFGE, antimicrobial resistance profiling, and composite analysis was calculated using the numerical index of discrimination (*D*) according to the method of Hunter and Gaston [60]. The discriminatory index represents the probability that two unrelated strains sampled from the test population will be placed into different typing groups [60].

## Abbreviations

C: ciprofloxacin; *D*: diversity index; E: erythromycin; FDA: Food and Drug Administration; Fla: flagellin; K: kanamycin; MIC: minimum inhibitory concentration; N: nalidixic acid; NARMS: National Antimicrobial Resistance Monitoring System; PFGE: pulsed-field gel electrophoresis; S: streptomycin; T: tetracycline.

## Authors' contributions

CML and JSS isolated and characterized the campylobacters recovered from poultry; EML carried out the antimicrobial resistance assays and molecular analysis; JMM carried out molecular and software analysis. All authors read and approved the final version of the manuscript.
